# Endoscopic Assistance in Minimally Invasive Repair of Acute Achilles Ruptures: A Prospective Observational Study Comparing Endoscopic-Assisted, Minimally Invasive, and Open Techniques

**DOI:** 10.3390/jcm14228117

**Published:** 2025-11-17

**Authors:** Paolo Ceccarini, Marco Donantoni, Lorenzo Lucchetta, Andrea Marinozzi, Pierluigi Antinolfi, Giuseppe Rinonapoli, Auro Caraffa

**Affiliations:** 1 Orthopaedic and Traumatology Unit, Department of Medicine, University of Perugia, 06156 Perugia, Italy; paoloceccarini84@gmail.com (P.C.); drlorenzolucchetta@pm.me (L.L.);; 2 Department of Orthopaedic and Trauma Surgery, Fondazione Policlinico Campus Bio-Medico, Via Alvaro del Portillo, 200, 00128 Rome, Italy; a.marinozzi@policlinicocampus.it; 3 Research Unit of Orthopaedic and Trauma Surgery, Università Campus Bio-Medico di Roma, Via Alvaro del Portillo, 21, 00128 Rome, Italy

**Keywords:** Achilles tendon rupture, endoscopic control, minimally invasive Achilles repair, percutaneous Achilles repair, tendon disorders

## Abstract

**Background**: The optimal management of acute Achilles tendon (AT) ruptures remains debated. Historically, surgical treatment has been preferred over conservative management because of a lower risk of rerupture. In recent decades, minimally invasive (MIS) and percutaneous techniques have been introduced to reduce the complications associated with the traditional open surgery. Comparable clinical outcomes have been demonstrated, with a lower rate of general complications but a higher risk of sural nerve palsy. Endoscopic assistance has been finally proposed to improve intraoperative visualization and potentially decrease this risk, although its actual potential benefit remains uncertain. **Methods**: A prospective observational study including a total of 94 patients who underwent surgical treatment for acute subcutaneous AT rupture was conducted. 60 of the patients were male and 34 were female, with a mean age at the time of surgery of 42.5 [18–78] years. The patients were then divided into three groups according to the surgical procedure performed: endoscopic-assisted MIS technique (A, n = 30), Ma-Griffith minimally invasive technique (M-G/MISI) (B, n = 34) and traditional open surgery (C, n = 32). Same post-operative protocol. The mean follow-up was 32 months (24–60 months). Patients were evaluated by time taken to return to driving (RTD), to their usual work activities (RTW), and, in active patients, to sports activities (RTS) at a level comparable to that prior to the injury was recorded. Clinical outcomes were evaluated with Achilles tendon Total Rupture Score (ATRS) and Ankle Hindfoot American Orthopaedic Foot and Ankle Society (AH-AOFAS) scores. **Results**: Significant differences among groups were found for RTD (*p* = 0.001), RTW (*p* < 0.001), and ATRS (*p* < 0.001), while RTS (*p* = 0.46) and AOFAS (*p* = 0.41) did not differ significantly. Post hoc analyses showed that the M-G/MISI group achieved faster return to driving and work and higher ATRS compared with the open group (all *p* < 0.05). No significant differences were detected between M-G/MISI and endoscopic-assisted techniques. **Conclusions**: All three techniques achieved good functional outcomes and low complication rates. Endoscopic assistance allowed visualization of suture passage and confirmation of gap reduction, but no superiority was observed in clinical outcomes or sural nerve safety. The M-G/MISI approach was associated with faster return to activity and higher ATRS compared with open repair.

## 1. Introduction

The Achilles tendon (AT) is the largest tendon in the human body. The optimal treatment and rehabilitation approach for acute Achilles tendon rupture has yet to be definitively clarified. Management options for acute AT rupture include both nonoperative and surgical strategies [1,2]. Recent evidence have shown that conservative management is effective in selected patients when associated with early functional rehabilitation [3,4,5,6,7]. Historically, surgery was preferred due to lower rerupture rates but posed risks of complications such as infections [8,9,10]. Recent data demonstrate a statistically significant difference in rerupture rates—3.6% following operative repair versus 12.1% with nonoperative management—although conservative treatment is associated with lower risks of infection and a reduced overall complication rate compared with surgical approach [11].

Over the last decades, in order to reduce open surgery complication rates, less invasive surgical strategies have been developed [12,13]. MIS and percutaneous techniques have shown comparable outcomes to open surgery [14,15], with an overall reduction in complication rates [16] except for an increased risk of sural nerve palsy and postoperative palpable knot formation [17,18]. Currently, surgical treatment of acute AT ruptures is associated with good clinical outcomes and an acceptable complication rate, with both open and MIS repair techniques representing viable options for surgical management [19]. To further reduce the rate of complications, particularly those related to the limited visualization inherent to percutaneous and MIS techniques, endoscopic assistance has been proposed in these procedures [20]. Although some studies have reported improved clinical outcomes with the use of endoscopic assistance [21], evidence remains inconclusive regarding its ability to decrease complication [22,23]. This is a prospective observational study that aims to compare open surgery [24], the M-G/MISI approach [25,26,27], and endoscopic-assisted MIS techniques [28] to evaluate the different outcomes between surgical approach in acute AT ruptures, focusing on whether endoscopic assistance may offer significant advantages.

## 2. Materials and Methods

### 2.1. Patient Population and Criteria

**Figure 1:**
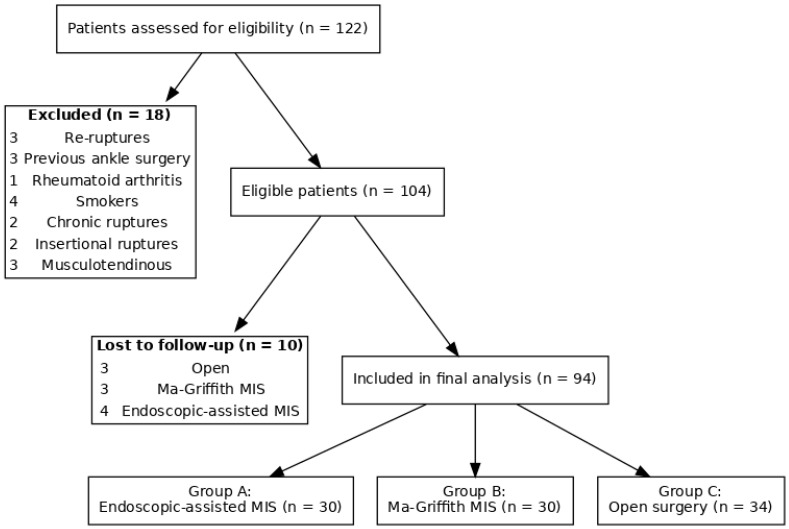
STROBE-style flow diagram of patient inclusion. From 122 patients assessed, 18 were excluded and 10 lost to follow-up, resulting in 94 patients allocated into three treatment groups.

This was a single-center prospective observational study conducted at the Department of Orthopaedics and Traumatology, University of Perugia. Patients who underwent surgical treatment for acute AT ruptures between December 2018 and January 2022 were assessed for eligibility. A total of 122 consecutive patients were initially identified. The study flow is summarized in the STROBE-style diagram; of 122 patients screened, 94 were included in final analysis (Figure 1). We included patients with acute subcutaneous AT rupture (operated within 4 weeks from trauma), aged over 18 years, of both genders. We excluded patients with insertional ruptures and musculotendinous junction tears, re-ruptures, chronic ruptures (>4 weeks), or significant previous ankle surgery. Patients with systemic diseases such as uncontrolled diabetes (HbA1c > 8%), rheumatoid arthritis undergoing treatment, smokers, infections, or neurological disorders were also excluded. From the initial 122 patients, 18 were excluded for not meeting the criteria and 10 were lost to follow-up. Our final cohort included 94 patients: 60 males and 34 females, with a mean age of 42.5 years (range 18–78). The patients were divided into three groups based on the surgical technique used:

- Group A: endoscopic-assisted minimally invasive technique.
- Group B: M-G/MISI.
- Group C: open surgery (Krackow suture).

Patients were evaluated with functional outcomes measured by the Achilles Tendon Total Rupture Score (ATRS) [29] and American Orthopaedic Foot and Ankle Society Ankle-Hindfoot Score (AOFAS).

In addition to clinical scores, return-to-activity outcomes were investigated: time to return to driving (RTD), time to return to daily work activities (RTW), and time to return to sport (RTS) was recorded.

Patients were evaluated at regular intervals, specifically at 2, 3, and 5 weeks postoperatively, and subsequently at 3, 6, and 12 months after surgery. RTD, RTW, and time to RTS were recorded during these visits, while functional outcomes were instead assessed at the final follow-up using the ATRS and the AH-AOFAS.

### 2.2. Statistical Analyses

Statistical analyses were performed using SPSS v23.0 (SPSS Inc., Chicago, IL, USA). Continuous variables are reported as mean ± standard deviation (SD), and categorical variables as absolute numbers and percentages. Data normality was assessed using the Shapiro–Wilk test. For normally distributed continuous variables, comparisons among the three surgical groups were performed using one-way ANOVA; for non-normally distributed data, the Kruskal–Wallis test was applied. When the overall test indicated statistical significance, pairwise post hoc comparisons were conducted using Welch’s *t*-tests with Bonferroni correction for multiple comparisons. Categorical variables were compared using the chi-square test or Fisher’s exact test, as appropriate.

Given the non-randomized, observational design, all between-group comparisons were considered exploratory, and results were interpreted as associations rather than causal relationships.

### 2.3. Surgical Techniques

All the patients of group A and B were operated on by a single surgeon, for group C there were also other orthopaedic surgeons working at the same Department of our Hospital.

#### 2.3.1. Group A—Endoscopic-Assisted Minimally Invasive Technique

The patient was positioned prone under peripheral nerve block anesthesia, with the foot hanging in plantar flexion. A thigh pneumatic tourniquet was inflated. A posterior distal-medial endoscopic portal was created to assess the lesion’s characteristics, including location, extension, and the condition of the peritenonium. The tendon defect was further confirmed by palpation, and four pairs of small (5 mm) skin incisions were made medial and lateral to the tendon edges, spaced 2 cm apart: two pairs at the lesion site and two in healthy tendon tissue at the proximal and distal stumps.

The peritenonium was detached at each incision level using small forceps. A modified M-G/MISI technique was performed using two non-absorbable sutures (Tycron or FiberWire No. 5). Sutures were first passed through the proximal stump incisions under arthroscopic guidance to ensure proper positioning, followed by passing sutures through the distal stump using the remaining incisions. The tendon stumps were approximated by tightening and knotting the sutures with the foot in 15–20° plantar flexion.

Endoscopy was used during the procedure to confirm tendon continuity and detect potential errors for immediate correction. An indirect sign of successful repair was the tendon obscuring the red light of the arthroscope camera. Finally, all incisions, including the arthroscopic portal and eight mini-portals, were closed with 3-0 resorbable sutures.

#### 2.3.2. Group B—Ma-Griffith with Minimally Invasive Small Incision (M-G/MISI) Technique

The patient was positioned prone under peripheral nerve block anesthesia, with the foot hanging in plantar flexion. A thigh tourniquet was inflated, and the tendon defect was identified by palpation. Three pairs of small (5 mm) incisions were made medial and lateral to the tendon edges, spaced 2 cm apart: one pair at the lesion site and two pairs in healthy tendon tissue at the proximal and distal stumps. To minimize sural nerve injury risk, the proximal stump incisions were made closer together.

The peritenonium was detached at each incision, and a modified M-G/MISI technique was performed using two non-absorbable sutures (ycron^®^ or FiberWire^®^ no. 5 (Tycron^®^, Ethicon Inc., Somerville, NJ, USA; FiberWire^®^, Arthrex Inc., Naples, FL, USA). Sutures were first passed transversely through the proximal and distal stump incisions. A midline incision at the lesion site provided direct visualization for stronger, more precise suturing. The tendon stumps were then tightened and knotted with the foot in 15–20° plantar flexion. Finally, all skin incisions were closed with 3-0 resorbable sutures.

#### 2.3.3. Group C—Open Surgery (Krackow Suture)

The patient was placed in a prone position, under peripheral nerve block anesthesia and with the foot hanging out the operation table in plantar flexion. A pneumatic tourniquet at the thigh was inflated. The tendon defect was then identified by palpation, and a longitudinal skin incision, based on the size of the gap and measuring approximately 6 to 8 cm, was performed medially to the tendon. After careful soft tissue dissection, the tendon stumps were visualized and then were sutured with different non-absorbable sutures (Tycron or Fiberwire n°5), depending on surgeon preference, using the Krackow technique. 44 wound closure in layers was then performed with non-absorbable stitches.

### 2.4. Post-Operative Protocol

The post-operative protocol remained uniform across all groups: a cast with the foot in 20° of plantar flexion was applied to the operated leg and maintained for 3 weeks, during which weight-bearing was prohibited. Suture removal occurred at 2 weeks, while the cast was replaced with a Walker orthosis set at 90° of flexion for an additional 2 weeks of non-weight bearing. Subsequently, patients were advised to wear comfortable shoes with heel support for one month. Partial weight-bearing was initiated after five weeks, gradually increasing until full weight-bearing was achieved, typically by the eighth week. Passive ankle flexion-extension exercises were permitted at 3 weeks, with caution to avoid forced dorsiflexion during the initial 2 weeks. Thereafter, active and active-assisted mobilization were encouraged, along with strengthening of leg musculature and calf muscle stretching. Weight bearing was allowed after a mean of 36.8 days (25–80) for group A, 37.4 days (28–60) for group B and 38 days (30–120) for group C.

## 3. Results

A total of 94 patients were included in the analysis: 30 in the endoscopic-assisted group (A), 30 in the M-G/MISI group (B), and 34 in the open group (C). Surgical approach was determined by the on-duty orthopedic surgeon based on pre-assigned rotation schedules. No randomization was performed. The groups did not differ significantly in demographics or clinical variables (Table 1).

The mean surgery time was 42.5 min for Group A, 33.3 for Group B, and 42.5 for Group C. Mean follow-up was 32 months (range 24–60).

No significant differences (*p* > 0.05) among the groups were found for mean surgery time, return to weight bearing on the injured leg and other descriptive variables.

### 3.1. Return to Drive, Work Activities, and Sport Activities

The average time to RTD was 72.1 days for group A, 66 days for group B and 82.3 days for group C. ANOVA showed a significant difference among groups (*p* = 0.001). Post hoc analysis demonstrated that patients in the M-G/MISI group RTD significantly earlier than those in the open group (*p* = 0.002); differences between the M-G/MISI and endoscopic-assisted groups were not significant (*p* = 0.16).

The mean time to RTW was 44.2 days for group A, 35.6 days for group B, and 51.1 days group C. A significant between-group difference was observed (*p* < 0.001). Post hoc testing confirmed a shorter RTW time in the M-G/MISI group compared with both group C (*p* < 0.001) and group B (*p* = 0.04) groups.

Four patients belonging to group A did not RTS, 4 for group B, and 8 for group C. The rest of the patients returned to their sports activity after a mean time of 202.8 days for group A, 208 days for group B and 219 days for group C (Table 2). The statistical analysis revealed no statistically significant differences (*p* > 0.05).

### 3.2. Clinical Outcomes: ATRS and AH-AOFAS

Mean ATRS was 79.3 ± 11.2 for group A, 87.6 ± 9.1 for group B and 72.75 ± 13.4 for group C. ANOVA reported a significant difference (*p* < 0.001) between groups and post hoc comparisons showed higher ATRS in the M-G/MISI group compared with both the open (*p* < 0.001) and endoscopic-assisted (*p* = 0.01) groups. The difference between endoscopic-assisted and open repairs was not significant (*p* = 0.08).

Mean AH-AOFAS was 96.2 ± 6.6 for group A, 96.4 ± 5.3 for group B and 94.0 ± 7.3 for group C (Table 3). No statistically significant differences (*p* = 0.41) were observed between the groups for AH-AOFAS.

### 3.3. Complications

Regarding the major complications in only 1 case (1%) we reported a DVT, and 2 patients (2%) needed a second surgery because of infection. No case of neurotmesis or axonotmesis was observed.

- Group A: 2 patients presented minimal paresthesia at the surgical site, resolved after three months conservatively. One patient with paresthesia developed a DVT and was treated with low molecular heparin.
- Group B: 4 patients complained of paresthesia at the surgical site, 2 patients complained of ankle stiffness. No major complication was reported.
- Group C: Two patients needed revision surgery because of infection with associated failure of the tendon suture, 20% suffered from postoperative ankle stiffness (Table 4).

No significant differences in complication rates emerged among the surgical techniques (*p* < 0.05).

## 4. Discussion

As stated in the introduction, this study aimed to compare the clinical outcomes of open surgery, the M-G/MISI approach, and endoscopic-assisted percutaneous repair for acute AT ruptures, focusing on whether endoscopic assistance may offer significant advantages. The surgical treatment of AT ruptures remains a subject of debate and controversy regarding the best approach. The risk of recurrence and wound complications has led to the increasing adoption of MIS techniques over open surgery, which nonetheless continues to deliver excellent outcomes. In fact, a recent meta-analysis involving 1465 patients found no difference in the rerupture rate when comparing open repair with minimally invasive surgery [15]. This meta-analysis, however, also indicates that MIS is associated with a higher risk of potential sural nerve injury. For this reason, endoscopic assistance has been proposed for percutaneous techniques to address these concerns. Based on the results of our study, endoscopic assistance did not lead to better outcomes or fewer complications to justify its routine use in clinical practice, considering the limitations related to cost, patient selection, and learning curve. In the current literature, studies on this topic are limited and consistent with our findings, showing no clear advantages from the use of endoscopic assistance other than providing direct visualization of suture passage and confirmation of tendon gap reduction [21,22,30,31].

In addition to these findings, our study also showed that all three surgical approaches achieved satisfactory functional results and low complication rates. The M-G/MISI technique was associated with faster return to driving and work and higher ATRS compared with open repair, while return to sport and AOFAS did not differ significantly among groups. In the current literature [32,33], comparative studies and available randomized controlled trials [34,35] evaluating open and minimally invasive techniques have shown largely comparable results, with specific advantages associated with each approach [36], as previously highlighted. Based on our findings, although the limitations of this study must be acknowledged, a trend toward superiority of minimally invasive techniques was observed for selected clinical outcomes. Differences in RTD, RTW, and ATRS were statistically significant in favor of the minimally invasive groups, whereas for RTS and AH-AOFAS, despite the absence of statistical significance, the values in Groups A and B were higher. This trend is consistent with the meta-analysis by Grassi et al. [14], which reported better superiority with MIS techniques to achieve better outcomes, and with the findings of Henrìquez et al. [37], who observed improved results at two-year follow-up in patients treated with MIS repair. At present, however, it is not possible to establish a clear superiority between the two surgical approaches, and both are considered valid and effective options for the management of acute AT ruptures [19].

### Challenges and Limitations

- Learning Curve and Cost Implications

Mastering endoscopic techniques requires specialized training, which may limit its availability in some settings. However, with advancements in technology and training programs, this gap is likely to narrow over time.

The equipment and expertise required for endoscopic repair can increase costs compared to traditional open repair. However, the potential for better long-term outcomes and fewer complications may offset these costs.

- Patient Selection

Endoscopic-assisted AT repair is not suitable for all patients, as factors such as tendon quality, rupture pattern, timing of surgery, and lesion location must be carefully evaluated. This technique is strongly recommended for non-insertional or muscle-tendon junctional ruptures. The method offers significant diagnostic advantages, ensuring safe suture passage, particularly through the proximal lateral portal, and confirming gap reduction and reinforcing the repaired site when needed. Its minimally invasive nature makes it an effective option for acute AT ruptures. However, success relies on surgeon expertise and proper patient selection. Further research and long-term studies are needed to refine the technique and establish its role as a standard treatment approach. Nevertheless, no major neurological complications were observed across our three groups. Despite this, sural nerve injury remains a potential complication of the procedure, even with endoscopic assistance [30]. Lui argues that while endoscopy assistance cannot reduce the risk of sural nerve injury, it can be helpful in achieving the suture passage in the proximal stump. Therefore, the future goal should be the development of an “all-inside” technique that avoids the posterolateral portal [23]. No significant differences were found concerning major complications. As already mentioned, open traditional surgery is generally associated with a higher risk of infection, while percutaneous surgery carries a potential greater risk of sural nerve damage [38]. In this study, no notable benefits were observed from using endoscopy assistance in MIS treatment as a compromise aimed at combining the advantages of both techniques, while eliminating or reducing the complications of each and, ultimately, leading to better clinical outcomes.

- Limitations

The main limitations of this study are the absence of randomization, blinding, and a non-injured control group, which reduce the strength of the findings and limit causal inference. Patient allocation depended on the on-duty surgeon, introducing potential selection bias; however, all participating surgeons had comparable experience with each of the techniques. In addition, no a priori power analysis was performed. Although a post hoc analysis was conducted, the lack of preplanned sample size calculation limits the statistical power and increases the risk of type II error. The analyses were exploratory and based on between-group comparisons without multivariable adjustment; therefore, the results should be interpreted as associative rather than causal. Despite these limitations, this study provides valuable comparative data on three distinct surgical approaches for acute AT ruptures. Future randomized and controlled studies with blinded assessment are warranted to confirm and expand these findings. The generalizability of the present results is inherently limited by the single-center design and relatively small sample size; therefore, validation through larger, multicenter investigations would be advisable

## 5. Conclusions

Endoscopic assistance in acute AT repair allowed better visualization of suture passage and confirmation of tendon gap reduction. Based on the results of this study, no superiority was observed in terms of clinical outcomes or safety, including the risk of sural nerve injury. All three surgical approaches achieved good outcomes and low complication rates. The M-G/MISI technique was associated with faster return to driving and work, and higher ATRS compared with open repair, while return to sport and AH-AOFAS did not differ significantly among groups.

Larger randomized controlled trials are needed to validate these results and clarify whether endoscopic assistance offers clinical advantages.

## Figures and Tables

**Table 1:** Demographic characteristics of our sample.

	Total	Group A	Group B	Group C
N° patients	94	30	30	34
Sex (M:F)	60:34	23:13	19:10	18:11
Age (years)	42.54 (18–78)	43.32 (24–66)	40.21 (18–69)	43.35 (22–78)
BMI (mean values)	26.63	26.54	25.00	27.12
Follow-up (months)	32 (24–60)	32 (24–56)	31 (24–60)	31 (24–58)

**Table 2:** Mean values of days spent to return to driving, return to work activities and return 2 to sports for each group.

	Group A	Group B	Group C	*p* Value
**RTD**	72.18 ± 15.9	66.00 ± 14.5	82.33 ± 18.1	*p* = 0.013
**RTW**	44.24 ± 11.1	35.64 ± 8.9	51.12 ± 12.8	*p* < 0.0001
**RTS**	202.83 ± 54.8	208.00 ± 56.2	219.00 ± 59.1	*p* = 0.46

**Table 3:** Achilles tendon total rupture score (ATRS) and American Orthopaedic Foot and Ankle Society Ankle-Hindfoot Scoring System (AH-AOFAS) mean score values stratified by groups.

	Group A	Group B	Group C	*p* Value
**ATRS score**	79.33 ± 11.2	87.64 ± 9.1	72.75 ± 13.4	*p* < 0.0001
**AH-AOFAS score**	96.28 ± 6.6	96.44 ± 5.3	94.02 ± 7.3	*p* = 0.41

**Table 4:** Total major and minor complications, stratified by groups.

	Total	Group A	Group B	Group C	*p* Value
Minor					
Post-op paresthesia	10	2	4	4	*p* = 0.67
Shoe discomfort	5	2	1	2	*p* = 0.83
Major					
DVT	1	1	0	0	*p* = 0.34
Infection	2	1	0	1	*p* = 0.61
Axono/neurotmesis	0	0	0	0	*p* = 0.43
Rate of reoperation	2	0	0	2	*p* = 0.16

## Data Availability

Data are fully available from the corresponding author upon request.

## Abbreviations

The following abbreviations are used in this manuscript:

AT	Achilles tendon
MIS	minimally-invasive surgery
M-G/MISI	Ma-Griffith minimally invasive small incision
BMI	body mass index
ATRS	achilles tendon total rupture score
AH-AOFAS	American Orthopaedic Foot and Ankle Society Ankle-Hindfoot Score
RTD	return to driving
RTW	return to work
RTS	return to sports
